# Apelin promotes osteosarcoma metastasis by upregulating PLOD2 expression via the Hippo signaling pathway and hsa_circ_0000004/miR-1303 axis

**DOI:** 10.7150/ijbs.77688

**Published:** 2023-01-01

**Authors:** Nguyen Thi Nha Trang, Chao-Yang Lai, Hsiao-Chi Tsai, Yuan-Li Huang, Shan-Chi Liu, Chun-Hao Tsai, Yi-Chin Fong, Huey-En Tzeng, Chih-Hsin Tang

**Affiliations:** 1 School of Medicine, China Medical University, Taichung, Taiwan.; 2 Department of Medical Laboratory Science and Biotechnology, Asia University, Taichung, Taiwan.; 3 Department of Medical Research, China Medical University Hospital, China Medical University, Taichung, Taiwan.; 4 Department of Medical Education and Research, China Medical University Beigang Hospital, Yunlin, Taiwan.; 5 Department of Orthopedic Surgery, China Medical University Hospital, Taichung, Taiwan.; 6 Department of Sports Medicine, College of Health Care, China Medical University, Taichung, Taiwan.; 7 Department of Orthopaedic Surgery, China Medical University Beigang Hospital, Yunlin, Taiwan.; 8 Department of Medical Research, Taichung Veterans General Hospital, Taichung, Taiwan.; 9 Division of Hematology/Medical Oncology, Department of Medicine, Taichung Veterans General Hospital, Taichung, Taiwan.; 10 Ph.D. Program for Cancer Molecular Biology and Drug Discovery, and Graduate Institute of Cancer Biology and Drug Discovery, College of Medical Science and Technology, Taipei Medical University, Taipei, Taiwan.; 11 Graduate Institute of Biomedical Sciences, China Medical University, Taichung, Taiwan.; 12 Chinese Medicine Research Center, China Medical University, Taichung, Taiwan.

**Keywords:** Apelin, PLOD2, metastasis, osteosarcoma

## Abstract

Osteosarcoma is a highly mortal bone tumor, with a high metastatic potential, promoted in part by the enzyme procollagen-lysine 2-oxoglutarate 5-dioxygenase 2 (PLOD2). Increasing level of PLOD2 in osteosarcoma tissue correlates with lymphatic and distant metastasis. The adipokine apelin (APLN) is also found in different cancers and APLN upregulation promotes angiogenesis and metastasis, but its effects on osteosarcoma metastasis are uncertain. We explored APLN functioning in metastatic osteosarcoma. An analysis of records from the Gene Expression Omnibus (GEO) database showed higher levels of APLN expression in osteosarcoma tissue than in normal tissue. Similarly, levels of *APLN* and *PLOD2* mRNA synthesis were upregulated in osteosarcoma tissue. Levels of APLN and PLOD2 protein correlated positively with osteosarcoma clinical stages. APLN increased PLOD2 expression in human osteosarcoma cell lines and cell migration via the mammalian Sterile 20-like kinase 1 (MST1), monopolar spindle-one-binder protein (MOB)1, and YAP cascades, and through hsa_circ_0000004 functioning as a sponge of miR-1303. We also found that knockdown of APLN antagonized lung metastasis in mice with osteosarcoma. APLN may be a therapeutic target in osteosarcoma metastasis.

## Introduction

Osteosarcoma is a mortal primary skeleton tumor derived from primitive bone-forming mesenchymal cells. Its high potential for metastasis is associated with poor survival rates 1. Osteosarcoma tends to metastasize to the lung, then to other organs in the body. The development of metastasis involves different steps of cancer cell dissemination from primary cancer, migration within the blood vasculature, and the build of clinically detectable pulmonary metastases 2. Around 15-20% of osteosarcoma patients display pulmonary metastases at diagnosis and approximately 40% of patients develop metastases at a later stage of the disease 3. The 5-year survival rate is 20% for patients who develop metastases 4. Even with optimal induction therapy, 30-40% of osteosarcoma cases with 80% of relapse and experience disease recurrence in the lungs. Novel therapies for metastatic osteosarcoma are required.

The transformation of the extracellular matrix (ECM) has a critical role in the proliferation and metastasis of osteosarcoma 5. Numerous ECM elements including fibronectin, proteoglycans, collagen, and laminins participate in osteosarcoma metastasis via intertwining and distinct mechanisms 6. Collagen, the main component of ECM, provides the scaffold for ECM assembly. In numerous cancer types, collagen is said to be the “highway” for cell motility 7. In human cancers, stabilized collagen accumulation is increased by diverse covalent collagen cross-linking 8. Collagen crosslinking and deposition rely on lysyl hydroxylation catalyzed by procollagen-lysine 2-oxoglutarate 5-dioxygenase (PLOD). In the PLOD family, PLOD2 is crucial for stabilizing collagen crosslinking formation through lysyl hydroxylase residues 9. PLOD2 modulates ECM by interacting with collagen fibers in the tumor matrix 10. The increased expression of PLOD2 is critical for the motility of tumors 11. PLOD2 is overexpressed in various cancers such as oral carcinoma 12, glioblastoma 13, bladder cancer 14, bone metastasis 15, and sarcoma 16, and is correlated with a poor prognosis. In non-small cell lung cancer, PLOD2 enhances metastasis by increasing cancer cell migration and inducing the reorganization of collagen 17. However, the functions and regulatory mechanisms of PLOD2 have not been elucidated in osteosarcoma metastasis.

Apelin (APLN), an adipokine, secreted by adipose tissue 18. In humans, APLN is a peptide containing a 77-amino acid preproapelin precursor encoded by the *APLN* gene 19. APLN is involved in different biological activities when it exists in various molecular forms. Apelin and APJ receptor systems are related to various physiological processes, such as angiogenesis 20, regulation of blood pressure 21, and energy metabolism 22. APLN contributes to pathological processes, including obesity, heart attacks, diabetes, and cancer 23. The function of APLN in tumor development and metastasis can be elucidated by the regulation of mediators controlled in tumor initiation and metastasis. APLN is a potent angiogenic factor in the development of blood vessels, endothelial cell proliferation, and migration *in vivo* studies 24, 25. APLN can induce tumor growth and proliferation of different cancers, including non-small cell lung cancer 26, ovarian cancer 27, and cholangiocarcinoma 28. APLN also stimulates cell migration in oral squamous cell carcinoma 29, human lung adenocarcinoma 30, and gastric cancer 31. These findings indicate that APLN plays a critical role in cancer metastasis. However, the effect of APLN in osteosarcoma metastasis is uncertain. This study therefore investigated whether APLN affects osteosarcoma migration and examined the involvement of signaling pathways.

## Materials and methods

Materials and methods relating to migration assay, western blot assay, reverse transcription-quantitative PCR (RT-qPCR) assay, immunohistochemistry (IHC) staining, cell transfection and RNA pull-down assay are all obtainable within supplementary information.

### Cell culture

Human osteosarcoma cell lines 143B and MG-63 were supplied by the Bioresource Collection and Research Center (BCRC) (Hsinchu, Taiwan). Both cell lines were cultured in Dulbecco's Modified Eagle's Medium (DMEM) medium complemented with 10% fetal bovine serum (FBS) and antibiotics, then sustained in an incubator at 37 °C in a moist atmosphere with 5% CO_2_.

### Analysis of mRNA expression profiles from the Gene Expression Omnibus (GEO) and The Cancer Genome Atlas (TCGA) database

Screening of GEO datasets indicated two microarrays associated with osteosarcoma (GSE16088 and GSE12865). The GSE16088 dataset contained numerous gene expression profiles from 14 human osteosarcoma tumor tissues and 6 normal bone tissues. The GSE12865 dataset contained gene expression profiles of 12 osteosarcoma tumor samples and 2 normal human osteoblast samples 32, 33. The Transcriptome profiles of osteosarcoma in the TCGA database were downloaded using UCSC Xena (http://xena.ucsc.edu). The 88 osteosarcoma samples that had undergone RNA-Seq analysis were used to examine the gene expression profiles of *APLN* and *PLOD2*.

### Luciferase activity assay

Luciferase plasmids containing the wild-type (WT) and mutant (MUT) sequences of the PLOD2 three prime untranslated region (3'-UTR) and hsa_circ_0000004 binding sites for miR-1303 were purchased by MDBio Inc. (Taipei, Taiwan). These recombinant plasmids were transfected with Lipofectamine 2000 and the 143B cell line for 24 h, then treated with APLN for 24 h. Luciferase activity was examined by the method described in our previous reports 34, 35.

### Chromatin immunoprecipitation-qPCR assay (ChIP-qPCR)

Osteosarcoma 143B cells were transfected with MST1 siRNA and MOB1 siRNA using Lipofectamine 2000 for 24 h, then treated with APLN for 24 h. This assay was performed as previously described 36, 37. DNA immunoprecipitated by YAP antibody was purified. The DNA pellet was subjected to qPCR to determine PLOD2 expression. The forward primer 5'AGCAAACAGTCCAGACGTGG 3' and the reverse primer 5'AGACAGGGATTCCAGGGGTG 3' were specifically designed to amplify across the PLOD2 promoter region (-499 to +100) containing the TEAD binding site. The input was used to perform qPCR from chromatin pellets prior to immunoprecipitation.

### Immunoprecipitation assay (IP assay)

The 143B cells were stimulated with APLN (3 ng/mL) for the indicated time interval. The cells were lysed in RIPA lysis buffer. A total of 50 µL of the cell lysates were used for the Western blot input assay, and the remaining lysates were incubated with 5 µL of 14-3-3 antibody at 4 °C for 2 h, then 10 µL of protein G agarose beads (Millipore, MA, USA) was added and mixed at 4 °C for 24 h. The immunoprecipitated complex was collected and washed twice with lysis buffer, then 5X Sample buffer was added and subjected to immunoblotting with YAP, and 14-3-3 antibodies.

### *In vivo* metastasis model

Balb/c nude mice were divided into two groups: the 143B/Luc (n=5) or 143B/shAPLN-Luc (n=5) group. The mice were provided with standard corncob litter in each cage; food and water were provided *ad libitum*. Before the injection of osteosarcoma cells, mice were exposed to 100% oxygen containing 3% isoflurane in a closed chamber for 3-5 min. After losing the righting reflex, the mice were transferred to a heating pad maintained at 36.5 °C to minimize pain and distress during the injection. The mice were injected in the lateral tail vein with a unit suspension containing 10^6^ cells (143B/Luc or 143B/shAPLN-Luc) in 200 μL PBS. All mice were housed under a 12/12 h light/dark cycle and survival was checked daily. The development of lung metastasis was monitored by bioluminescence imaging (Xenogen IVIS imaging system). A 9 weeks post-injection, the mice were sacrificed by CO_2_ inhalation. The lungs were removed and photographed, then fixed in 10% formalin, embedded in paraffin, and processed for IHC staining with APLN and PLOD2 antibodies. All animal experiments were conducted in accordance with a protocol approved by the Institutional Animal Care and Use Committee of China Medical University.

### Statistical analysis

All values are expressed as the mean ± standard deviation (SD). Differences between experimental groups and controls were assessed by the Student's *t-*test. One-way analysis of variance (ANOVA) was performed for statistical analyses of multiple groups. Statistical differences were considered significant if the *p-*value was <0.05.

## Results

### Clinicopathological characteristics of APLN in human osteosarcoma tissue

**Figure 1:**
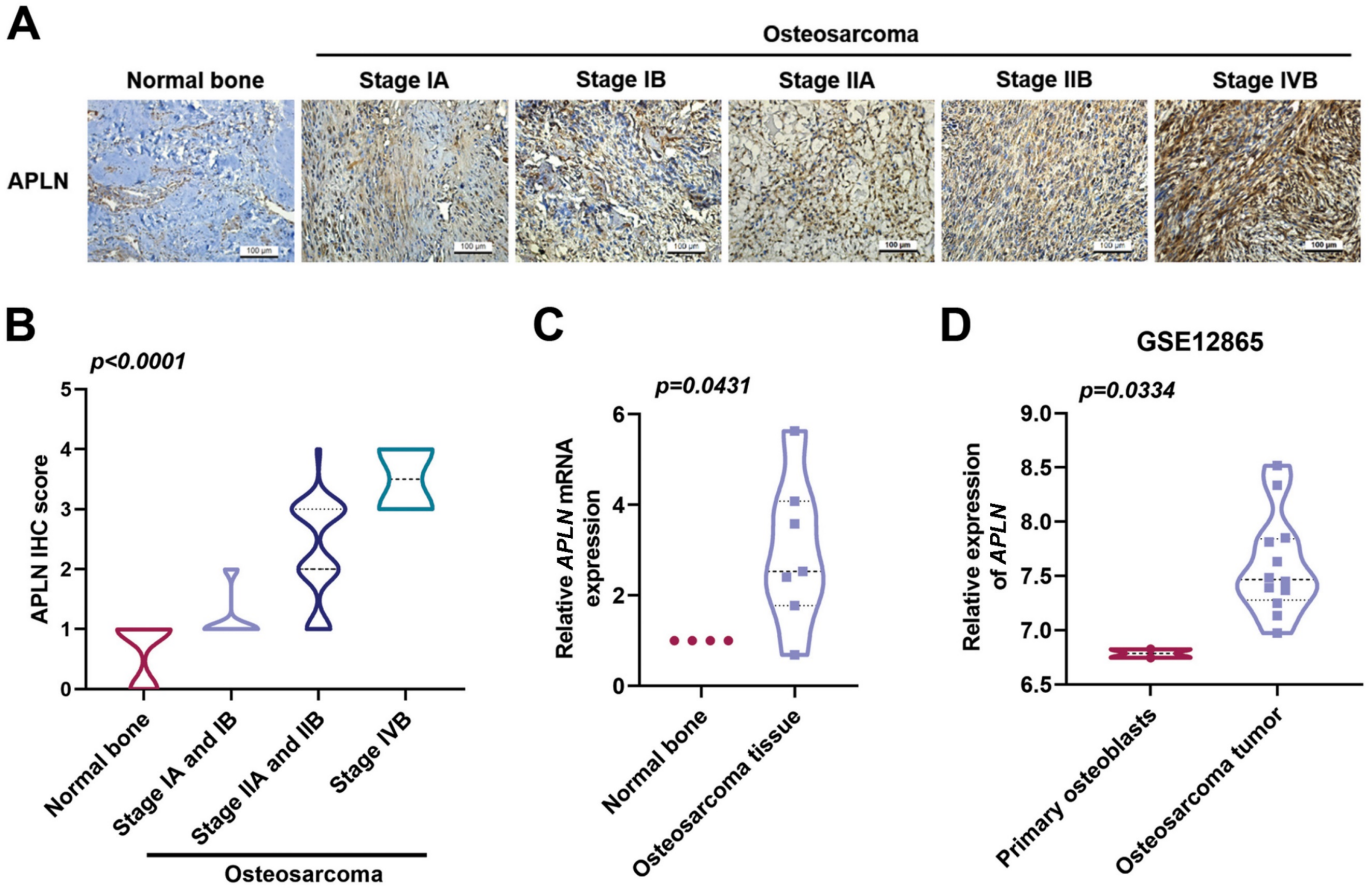
** Clinicopathological characteristics of APLN in human osteosarcoma tissue. (A-B)** Normal bone and osteosarcoma specimens were subjected to IHC staining with APLN antibody (scale bar 100 µm), then quantified.** (C)** *APLN* mRNA expression in normal bone and osteosarcoma tissue were examined by qPCR.** (D)** *APLN* mRNA expression in primary osteoblast and osteosarcoma tissue was analyzed in samples from the GEO GSE12865dataset.

APLN level is closely associated with the progression of many cancers and poor clinical outcomes 26. The levels of APLN expression were significantly associated with osteosarcoma clinical stages (Figure 1A-B). In addition, levels of APLN expression in osteosarcoma tissue were higher than in normal bone tissue (Figure 1C). Similarly, the analysis of osteosarcoma tissue samples from the GEO dataset (GSE12685) showed that levels of *APLN* mRNA expression were higher in osteosarcoma tissue than in primary osteoblasts (Figure 1D). These results revealed that APLN is upregulated in osteosarcoma and positively associated with osteosarcoma clinical stages.

### Levels of APLN and PLOD2 expression are positively correlated in human osteosarcoma tissue

**Figure 2:**
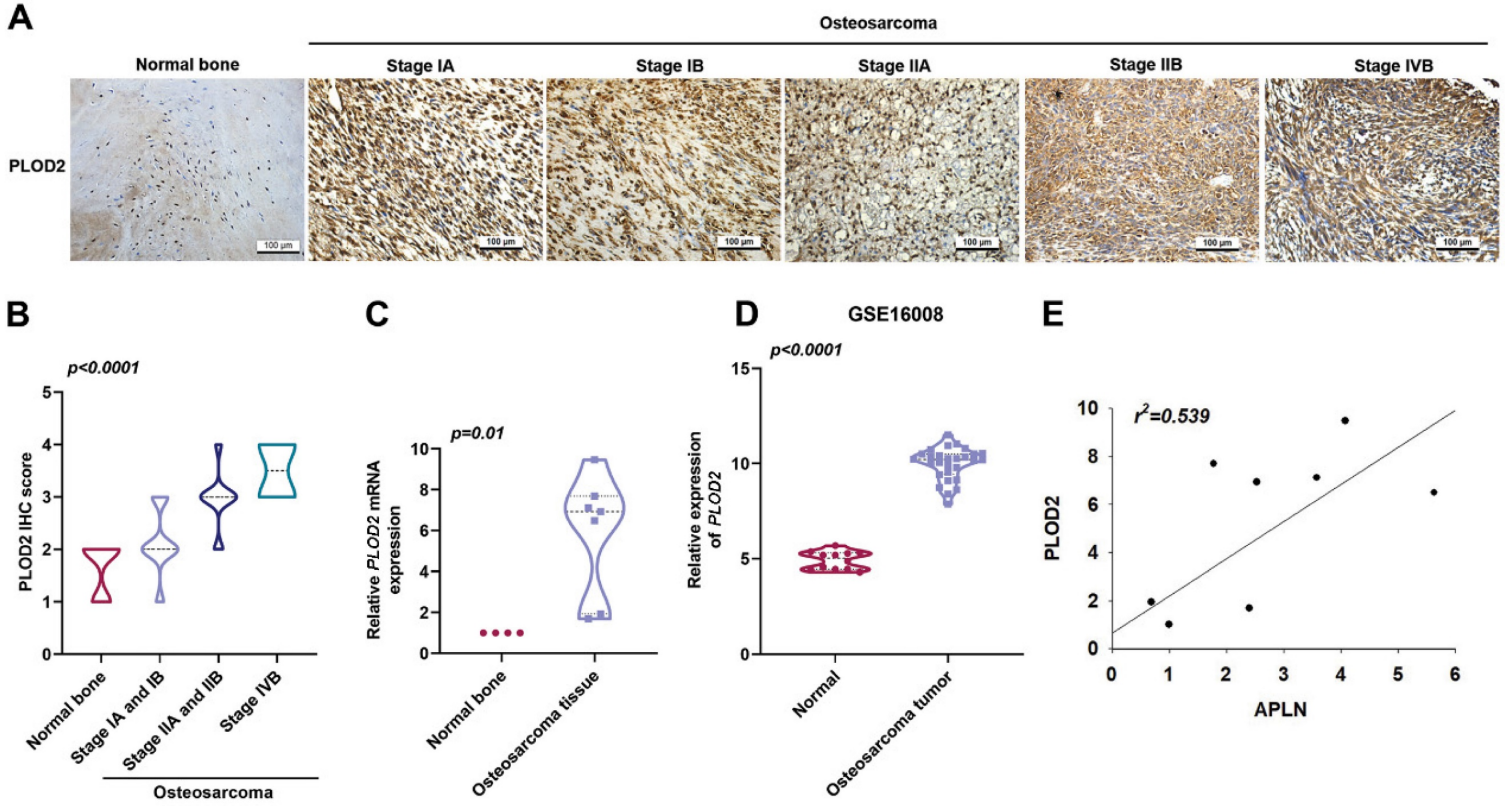
**A positive correlation between APLN and PLOD2 expression in human osteosarcoma tissue. (A-B)** Normal bone and osteosarcoma specimens were subjected to IHC staining with PLOD2 antibody (scale bar 100 µm), then quantified. **(C)** *PLOD2* mRNA expression in normal bone and osteosarcoma tissue were examined by qPCR. **(D)** *APLN* mRNA expression in normal and osteosarcoma tissue was analyzed in samples from the GEO GSE16008 dataset. **(E)** Analysis of correlations between APLN and PLOD2 expression in osteosarcoma tissue.

PLOD2 is a major enzyme in the modification of fibrotic collagen and its dysregulation enhances cancer progression metastasis 11. Upregulation of PLOD2 expression is associated with lymph node and pulmonary metastasis, as well as poor outcomes in osteosarcoma 38. IHC staining with PLOD2 antibody in osteosarcoma tissue revealed significant associations with clinical disease stages (Figure 2A-B). In addition, osteosarcoma tissue revealed upregulated levels of *PLOD2* mRNA expression compared to those in normal bone tissue (Figure 2C). Similarly, the GEO dataset (GSE16008) analysis indicated significantly higher *PLOD2* mRNA expression in osteosarcoma tissue compared with levels in normal bone tissue (Figure 2D). A positive correlation was found between APLN and PLOD2 expression in osteosarcoma tissue (Figure 2E). These findings demonstrated that the expression of both APLN and PLOD2 correlate positively with osteosarcoma clinical stages.

### APLN promotes osteosarcoma cell migration and increases PLOD2 expression in osteosarcoma tissue

**Figure 3:**
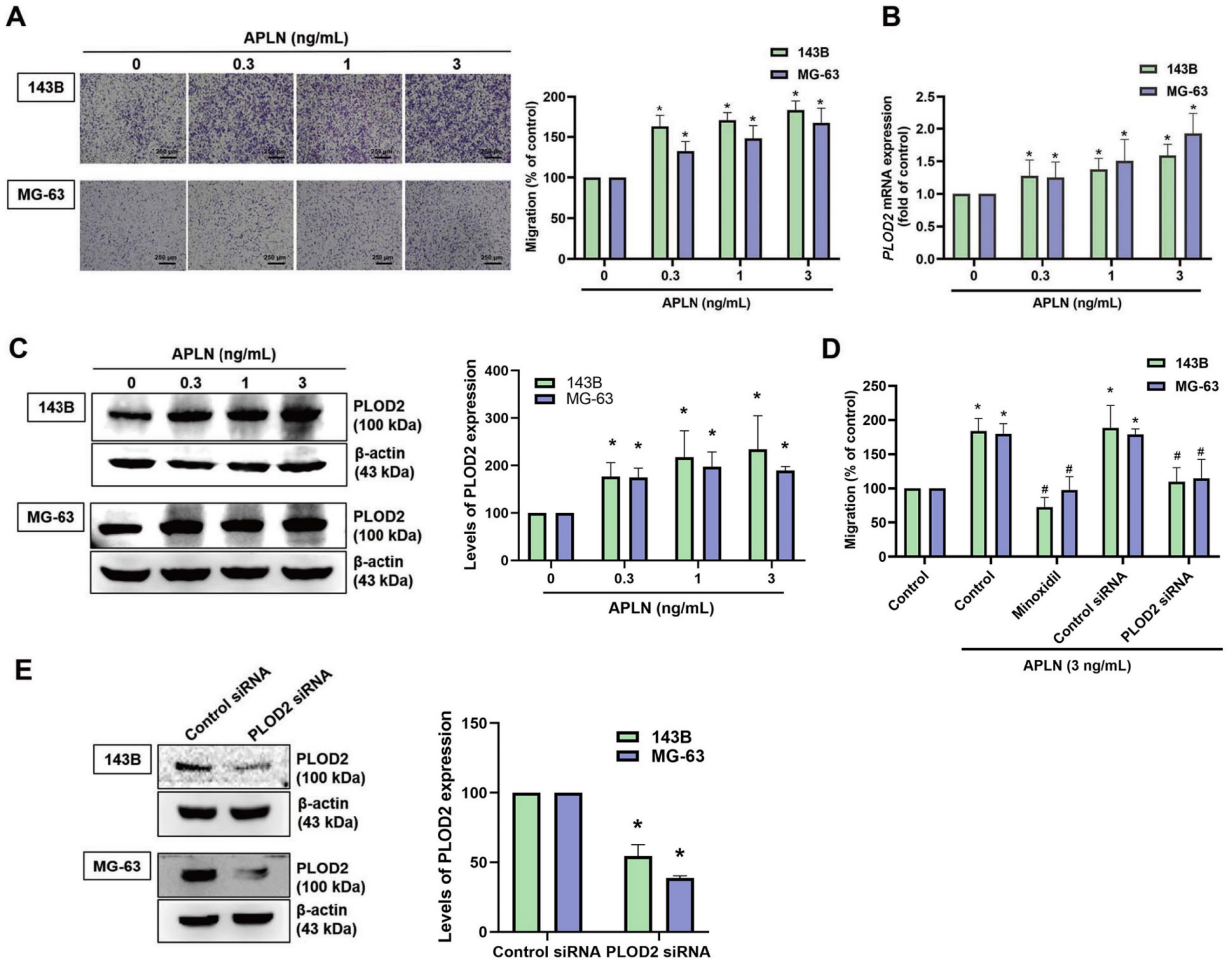
** Apelin promotes osteosarcoma cell migration by increasing PLOD2 expression. (A-C)** Osteosarcoma cells were treated with different concentrations of APLN (0, 0.3, 1, or 3 ng/mL) for 24 h. The Transwell assay, qPCR and Western blot examined *in vitro* migratory activity, and mRNA and protein expression of PLOD2, respectively. **(D)** Cells were pretreated with a PLOD2 inhibitor (minoxidil, 0.5 mM) or transfected with PLOD2 siRNA, then stimulated with APLN (3 ng/mL) for 24 h. The Transwell assay quantified *in vitro* migration.** (E)** Osteosarcoma cells were transfected with PLOD2 siRNA for 24 h and levels of *PLOD2* mRNA were assessed by qPCR. *p < 0.05 compared with controls; ^#^ *p <* 0.05 compared with the APLN-treated group.

APLN facilitates the migration of oral squamous cell carcinoma cells and gastric cancer cells 39. In this study, analyses of metastatic and nonmetastatic osteosarcoma tissue samples from the TCGA database revealed higher levels of *APLN* and* PLOD2* expression in the metastatic tissue samples than in the nonmetastatic tissue samples (Suppl. Fig. S1). We therefore first examined whether APLN induces osteosarcoma cell migration. APLN dose-dependently promoted migratory activity of osteosarcoma cells (Figure 3A). PLOD2 mediates tumor metastasis in various cancers and its dysregulation enhances cancer progression and metastasis 11, 40. We next investigated whether APLN affects PLOD2 expression in osteosarcoma cells. Stimulation of osteosarcoma cells (143B and MG-63) with APLN significantly increased PLOD2 mRNA and protein expression (Figure 3B-C). Pretreatment of cells with a PLOD2 inhibitor (minoxidil) or transfecting the cells with PLOD2 siRNA diminished APLN-promoted increases in migration (Figure 3D-E). These results indicate that APLN promotes cell migration by increasing PLOD2 expression in osteosarcoma.

### MST1/MOB1 signaling is involved in APLN-induced increases of PLOD2-mediated migration of osteosarcoma cells

**Figure 4:**
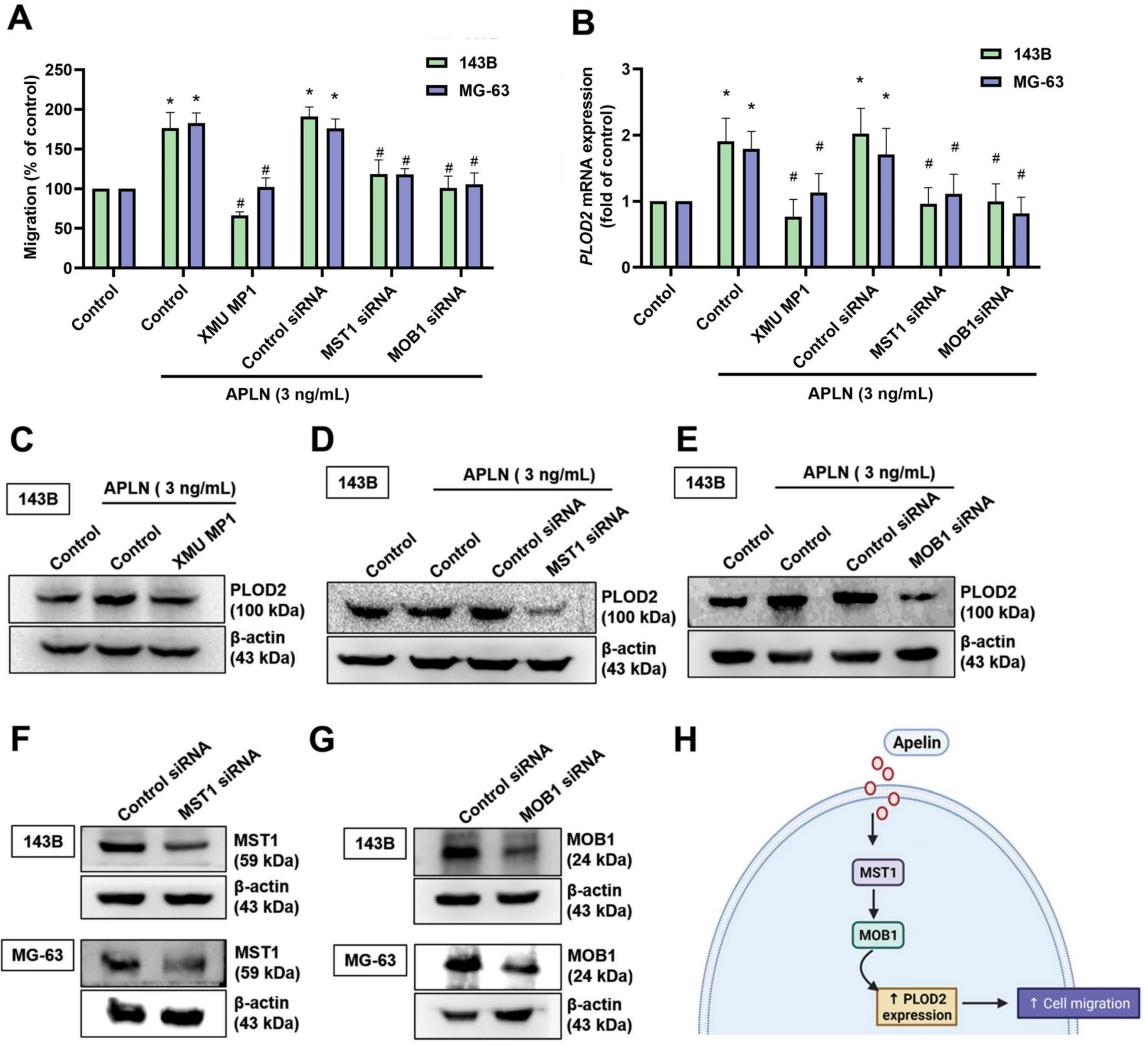
** The MST1/MOB1 pathway is involved in APLN-induced osteosarcoma cell migration and increases PLOD2 expression. (A-E)** Osteosarcoma cells were incubated with a MST1 inhibitor (XMU MP1, 3 µM) for 30 min, or transfected with MST1 or MOB1 siRNAs for 24 h, then treated with APLN (3 ng/ml) for 24 h. The Transwell, qPCR and Western blot assays examined *in vitro* migratory activity and levels of PLOD2 expression. **(F-G)** 143B and MG-63 cell lines were transfected with MST1 or MOB1 siRNAs for 24 h, then MST1 and MOB1 expression was examined by Western blot. **(H)** The schema illustrates the involvement of MST1 and MOB1 in APLN-induced PLOD2 expression and the migration of osteosarcoma cells. * *p <* 0.05 compared with controls; ^#^ *p <* 0.05 compared with the APLN-treated group.

Recent studies have reported that several signaling pathways (including the PI3K/Akt/mTOR, NF-κB, JAK/STAT, Hippo and hypoxia pathways) have an important role in cancer metastasis, including osteosarcoma 41-44. We examined whether these five signaling pathways mediate APLN-induced increases in PLOD2 expression and promote osteosarcoma cell migration. We found that verteporfin (an inhibitor of YAP and a transcriptional co-activator of the Hippo signaling pathway) significantly inhibited APLN-induced increases in *PLOD2* mRNA expression (Suppl. Fig. S2). We therefore suggest that the Hippo signaling pathway is involved in APLN-induced stimulation of osteosarcoma cell migration. The canonical mammalian Hippo pathway consists of MST1 and MOB1, and their dysfunction enhances cancer metastasis 45, 46. We examined whether APLN-induced promotion of osteosarcoma cell migration is mediated by MST1 and MOB1. Pretreating the cells with a MST1 inhibitor (XMU MP1) or transfecting them with MST1 and MOB1 siRNAs significantly inhibited the effects of APLN upon cell migratory activity, as well as levels of *PLOD2* mRNA and protein expression (Figure 4A-G). These results indicate that the MST1/MOB1 pathway regulates APLN-enhanced promotion of osteosarcoma cell migration and increases PLOD2 synthesis.

### YAP regulates APLN-induced increases in PLOD2 expression and osteosarcoma cell migration

**Figure 5:**
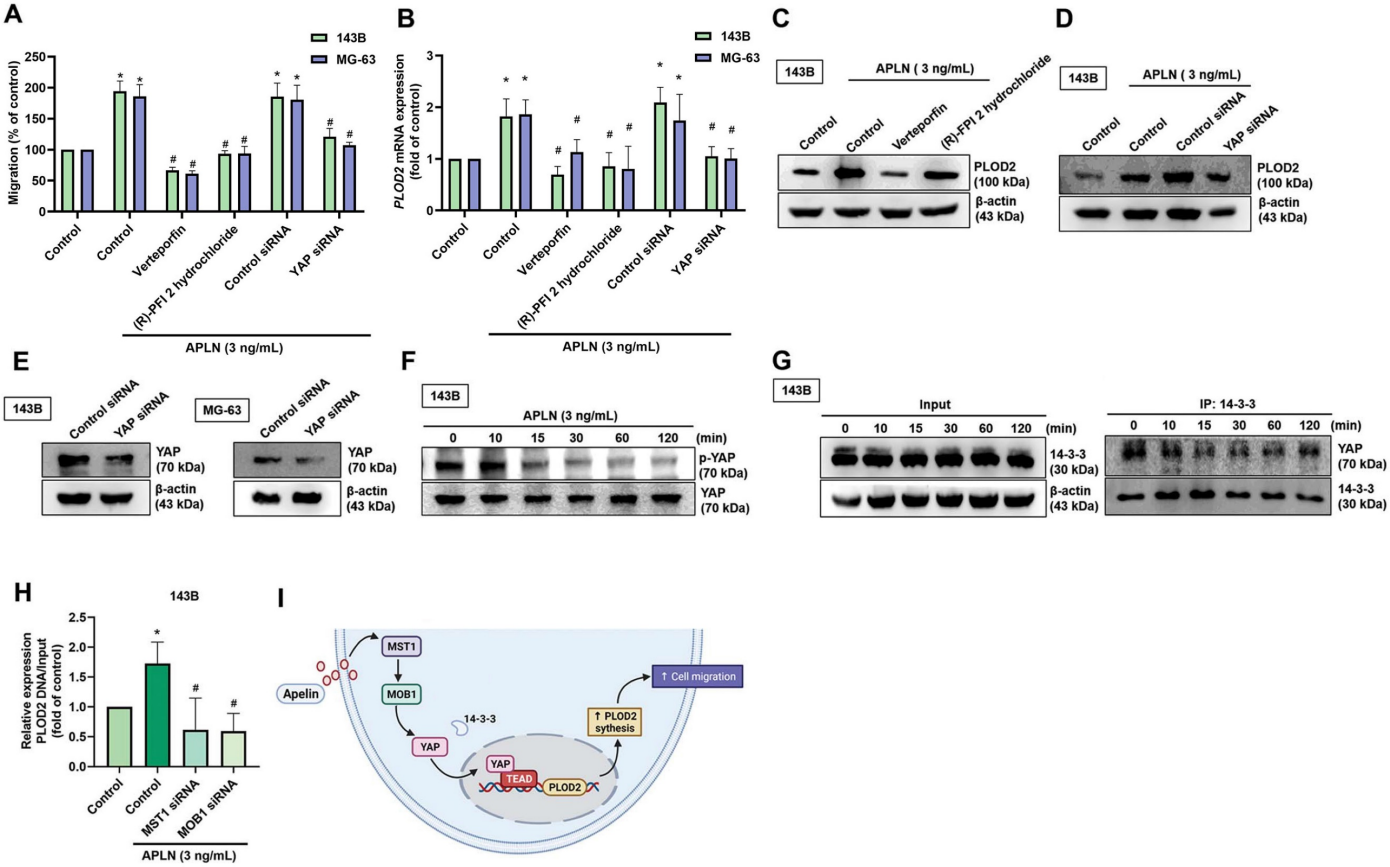
** The YAP pathway is related to APLN-induced osteosarcoma cell migration and increases PLOD2 expression. (A-D)** Osteosarcoma cell lines were treated with YAP inhibitors (verteporfin 0.5 µM or (R)-PFI 2 hydrochloride 10 µM) for 30 min or transfected with a YAP siRNA for 24 h, then stimulated with APLN (3 ng/mL) for 24 h. The Transwell assay, qPCR and Western blot determined *in vitro* migration, PLOD2 mRNA and protein expression, respectively. **(E)** 143B and MG-63 cell lines were transfected with a YAP siRNA for 24 h, then YAP expression was examined by Western blot. **(F)** 143B cells were treated with APLN (3 ng/mL) for the indicated times, and phosphorylation of YAP was examined by Western blot. **(G)** The immunoprecipitation assay determined the interaction between YAP and 14-3-3 in the 143B cell line treated with APLN (3 ng/mL) for the indicated times.** (H)** ChIP and qPCR were performed to verify the regulatory interaction of YAP and the PLOD2 promoter. **(I)** The schema illustrates how APLN-induced YAP nuclear localization promoted PLOD2 expression and increased osteosarcoma cell migration. Data are represented as means ± SD. * *p <* 0.05 compared with controls; # *p <* 0.05 compared with the APLN-treated group.

YAP is a major downstream effector of the Hippo pathway and its activation regulates target genes regulated to tissue growth and metastasis 47. We therefore examined whether YAP is involved in the roles of APLN upon cell migration and PLOD2 expression. Pretreating 143B and MG-63 cell lines with YAP inhibitors (verteporfin, and (R)-PFI 2 hydrochloride) or transfecting the cells with a YAP siRNA reduced the effects of APLN upon cell migration, as well as the levels of PLOD2 mRNA and protein expression (Figure 5A-E). APLN-induced reductions in YAP phosphorylation. (Figure 5F). By binding to YAP, 14-3-3 sequestered YAP in the cytoplasm, inhibiting the binding of YAP with transcriptional enhanced associate domain (TEAD) transcription factors 48. The result revealed decreases in the interaction between YAP and 14-3-3 when stimulated with APLN (3 ng/mL) for indicated times (Figure 5G). The transfection of 143B cells with MST1 and MOB1 siRNAs reduced APLN-enhanced binding of YAP to the PLOD2 promoter region (Figure 5H). These results indicate that YAP regulates APLN-induced increases in PLOD2 expression and migratory ability of osteosarcoma cells.

### APLN promotes PLOD2 expression and cell migration by downregulating miR-1303

**Figure 6:**
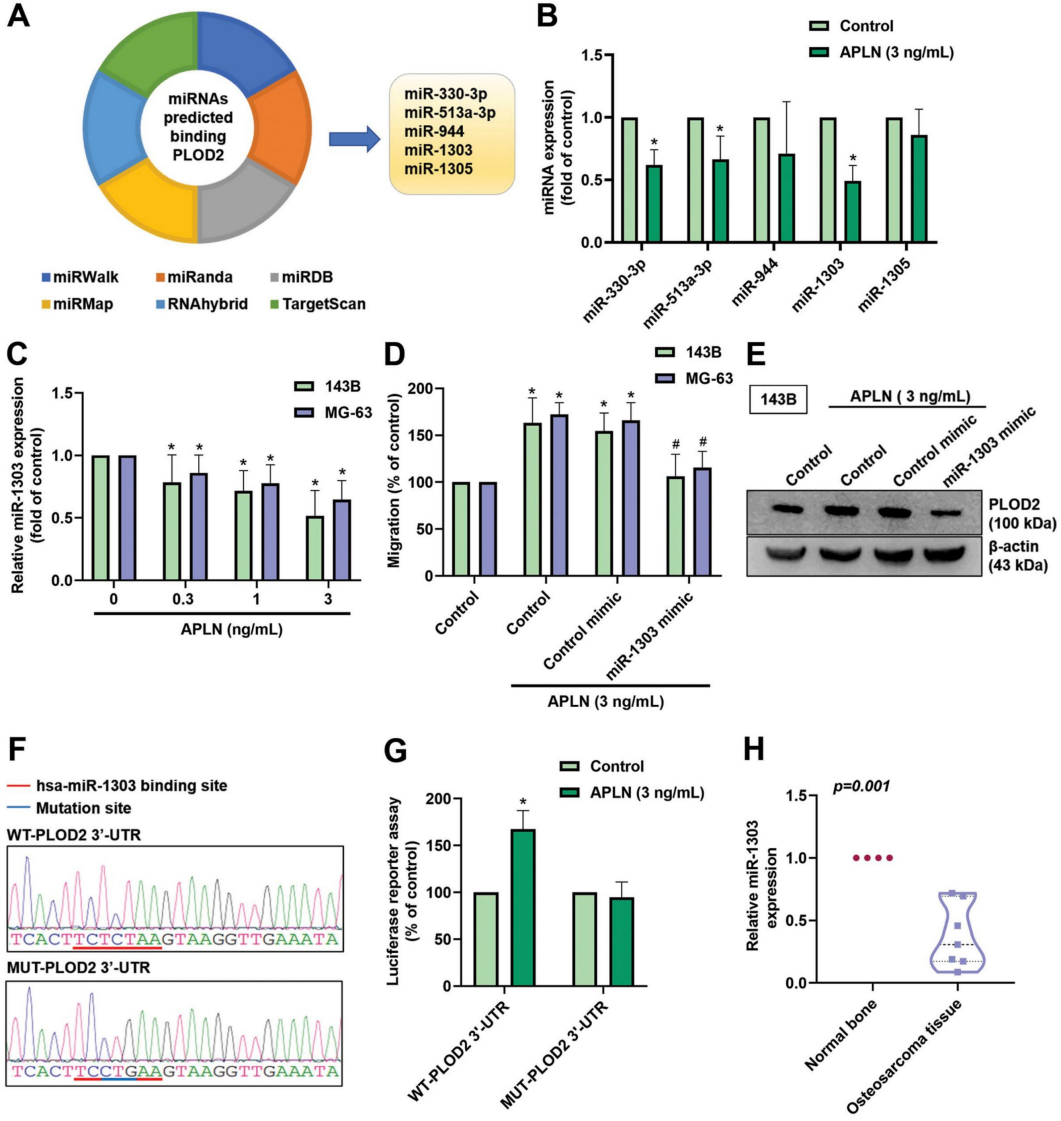
** APLN increases PLOD2 expression, which promotes osteosarcoma cell migration by downregulating miR-1303 expression. (A)** An analysis of 6 miRNA prediction databases pedicted 5 miRNAs that bind with PLOD2 (B) The 143B cell line was treated with APLN (3 ng/mL) and miRNAs were screened using a qPCR assay. **(C)** 143B and MG-63 cell lines were treated with different concentration of APLN (1, 0.3, 1, 3 ng/mL) for 24 h and miR-1303 expression was examined by qPCR. **(D-E)** Cells were transfected with miR-1303 mimic or control mimic for 24 hours, the treated with APLN (3 ng/mL) for 24 h. Transwell assay and Western blot examined the *in vitro* migration and PLOD2 protein expression. **(F)** The wild-type and mutant-PLOD2 3'-UTRs contained the miR-1303 binding site. **(G)** 143B cells were transfected with the WT-PLOD2 3'-UTR plasmid or MUT-PLOD2 3'-UTR plasmid for 24 h, then stimulated with APLN (3 ng/mL) for 24 h, and relative luciferase activity was measured. **(H)** Levels of miR-1303 expression in normal bone tissue and human osteosarcoma tissue were examined by qPCR. * *p <* 0.05 compared with controls; ^#^ *p <* 0.05 compared with the APLN-treated group.

MiRNAs are capable of modulating protein-coding genes by binding to the 3'-UTR of mRNA and play vital roles in oncogenesis and metastasis of human cancers 49, 50. The dysregulation of miRNAs affects angiogenesis induction, and invasion and metastasis activation 51. We first searched six online databases to identify miRNAs that may modulate PLOD2 expression. Based on overlapping records from the miRWalk, miRanda, miRDB, miRMap, RNAhybrid, and TargetScan databases, we found that 5 miRNAs (miR-330-3p, miR-513a-3p, miR-944, miR-1303, and miR-1305) bind to the 3'-UTR of PLOD2 (Figure 6A). Treatment of 143B cells with APLN (3 ng/mL) manifestly decreased the expression of miR-1303 (Figure 6B). Treatment of 143B and MG-63 cell lines with APLN (0, 0.3, 1, or 3 ng/mL) inhibited miR-1303 levels in a concentration-dependent manner (Figure 6C). MiR-1303 mimic abolished APLN-induced promotion of cell migration and PLOD2 expression (Figure 6D-E). Analyses of the PLOD2 3'-UTR luciferase plasmids found that APLN enhanced luciferase activity of the wild-type PLOD2 3′-UTR; APLN had no such effect upon the mutant PLOD2 3'-UTR (Figure 6F-G). MiR-1303 expression was manifestly downregulated in human osteosarcoma tissue compared with normal bone tissue (Figure 6H). These data demonstrate that APLN enhances PLOD2 expression and osteosarcoma cell migration by downregulating miR-1303 expression.

### APLN enhances PLOD2 expression and cell migration by regulating circ_0000004 sponging of miR-1303

**Figure 7:**
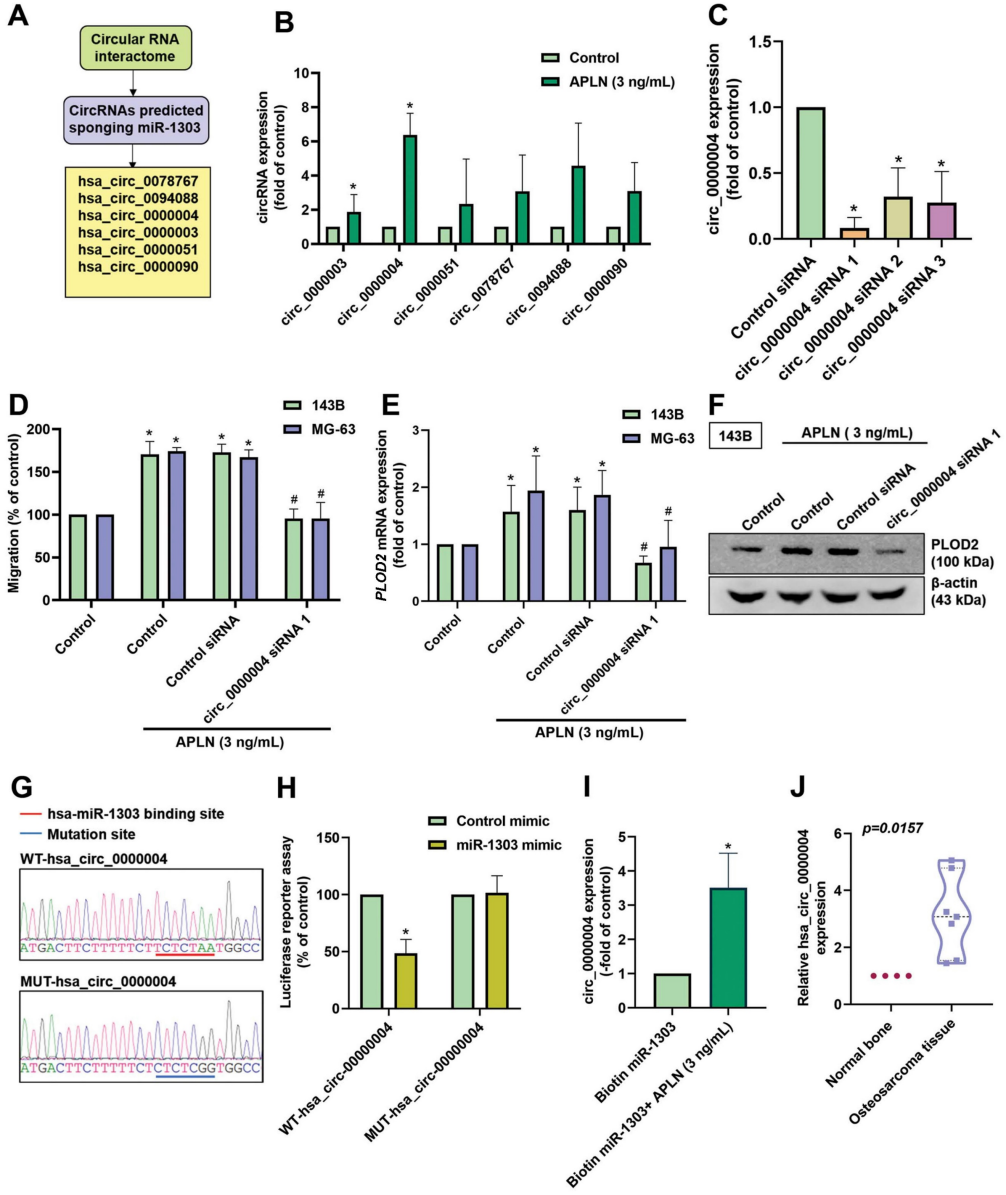
** APLN enhances osteosarcoma cell migration and increases PLOD2 expression by upregulating circ_0000004 sponging of miR-1303. (A)** circRNAs predicted by the Circular RNA Interactome to act as sponges of miR-1303. **(B)** The 143B cells were treated with APLN (3 ng/mL) and circRNAs were screened by qPCR. **(C)** 143B cells were transfected with 3 siRNAs of hsa_circ_0000004 for 24 h and hsa_circ_0000004 expression was examined by qPCR. **(D-F)** Osteosarcoma cells were transfected with circ_0000004 siRNA 1 for 24 h, then treated with APLN (3 ng/mL) for 24 h. The Transwell assay and Western blot examined *in vitro* cell migration, and PLOD2 mRNA and protein expression. **(G)** Schematic presents the hsa_circ_0000004 sequence containing the miR-1303 binding site.** (H)** 143B cell lines were transfected with WT-hsa_circ_0000004 plasmid or MUT-hsa_circ_0000004 plasmid, then transfected with control mimic or miR-1303 mimic for 24 h and relative luciferase activity was measured. **(I)** hsa_circ_0000004 was pulled down with biotin-labeled miR-1303 by the RNA pull-down assay. **(J)** Levels of miR-1303 expression in normal bone tissue and human osteosarcoma tissue were examined by qPCR. * *p <* 0.05 compared with controls.

CircRNAs (circular RNAs) play important functions in cancer progression and are recognized to act as sponges for miRNAs, leading to the inhibition of miRNA function 52, 53. Analysis of the Circular RNA Interactome predicted that 6 circRNAs (circ_0078767, circ_0094088, circ_0000004, circ_0000003, circ_0000051, and circ_0000090) bind to miR-1303 (Figure 7A). When we screened the expression of these circRNAs during APLN treatment (3 ng/mL) in osteosarcoma cells, hsa_circ_0000004 exhibited the highest expression (Figure 7B). To investigate the function of hsa_circ_0000004 in osteosarcoma cells, we designed 3 siRNAs of hsa_circ_0000004. Transfection of cells with these siRNAs significantly decreased hsa_circ_0000004 expression; hsa_circ_0000004 siRNA 1 had the greatest inhibitory effect (Figure 7C). Transfection of 143B cells with hsa_circ_0000004 siRNA 1 reduced APLN-induced promotion of cell migration, PLOD2 mRNA and protein expression (Figure 7D-F). To examine the interaction between hsa_circ_0000004 and miR-1303, we constructed luciferase reporter plasmids harboring wild-type-hsa_circ_0000004 (WT-hsa_circ_0000004) and mutant-hsa_circ_0000004 (MUT-hsa_circ_0000004) (Figure 7G). Luciferase activity of WT-hsa_circ_0000004 was inhibited by miR-1303, whereas MUT-hsa_circ_0000004 luciferase activity was not affected (Figure 7H). Results from the RNA pull-down assay indicated that a significant increase in hsa_circ_0000004 was pulled down by biotin-labeled miR-1303 when treated with APLN (3 ng/mL), confirming that hsa_circ_0000004 directly binds to miR-1303 (Figure 7I). Further analyses indicated the upregulation of hsa_circ_0000004 expression in human osteosarcoma tissue compared with normal tissue (Figure 7J). These results reveal that APLN promotes osteosarcoma cell migration and increases PLOD2 expression by upregulating hsa_circ_0000004 sponging of miR-1303.

### APLN knockdown inhibits osteosarcoma metastasis in a mouse model

**Figure 8:**
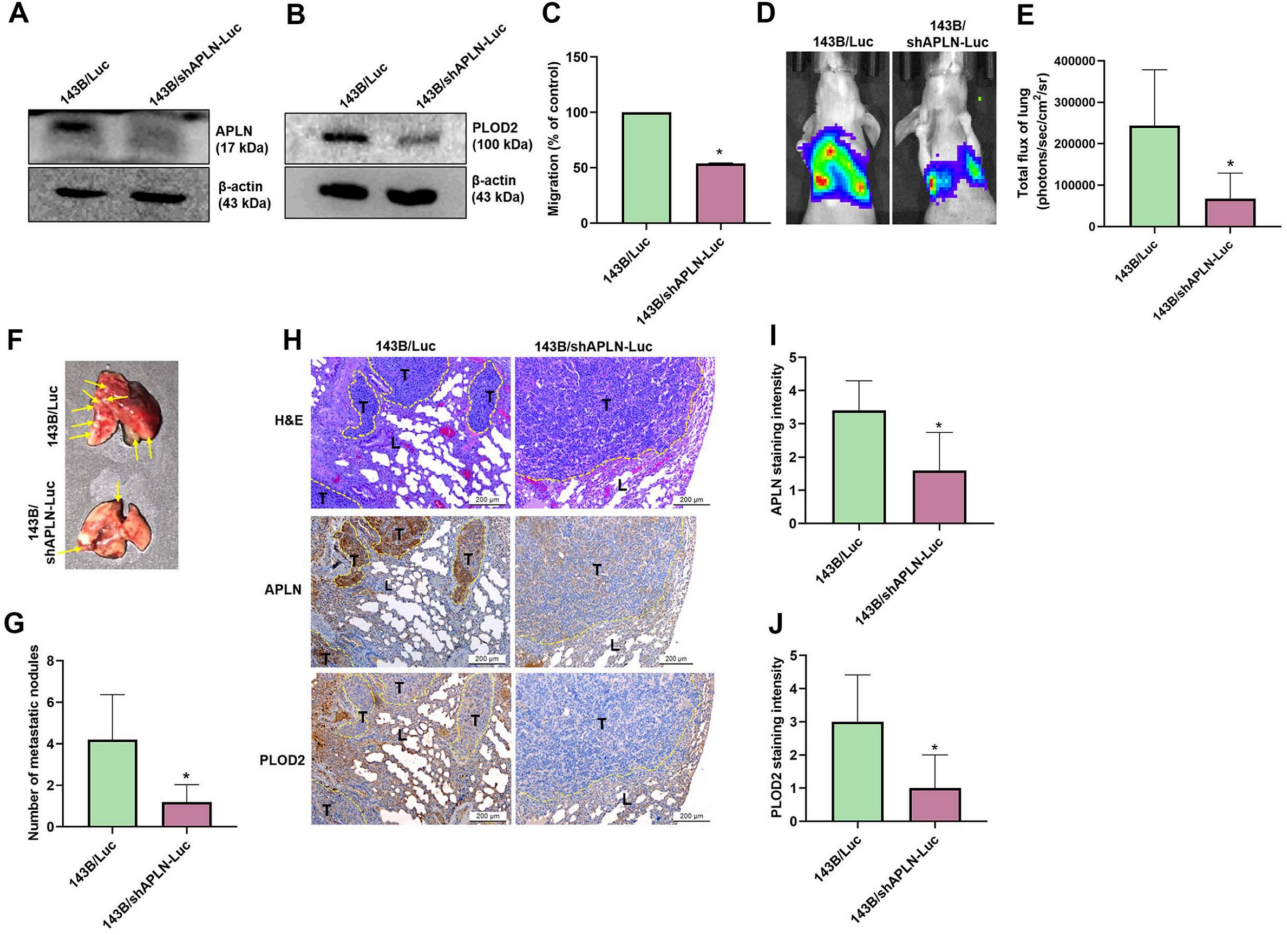
** Knockdown of APLN suppresses lung metastasis.** 143B/Luc cells stably expressing control-shRNA or APLN-shRNA were established. **(A-B)** APLN and PLOD2 protein expression was determined by Western blot. **(C)** Migration of 143B/Luc and 143B/shAPLN-Luc cells was examined by the Transwell assay. **(D)** 143B/Luc or 143B/shAPLN-Luc cells were injected into the lateral tail veins of Balb/c nude mice, and lung metastasis was monitored by bioluminescence imaging. **(E)** Quantification of *in vitro* bioluminescence imaging. **(F-G)** At 9 weeks after injection, the mice were sacrificed and the lung tissues were excised, photographed, and quantified for 143B/Luc and 143B/shAPLN-Luc expression. **(H)** IHC staining of APLN and PLOD2 expression in lung tumors. L, lung tissue; T, tumor.** (I-J)** Quantification of APLN and PLOD2 expression in IHC images. * *p <* 0.05 compared with 143B/Luc-control cells.

To confirm the effects of APLN upon osteosarcoma cell migration and PLOD2 expression *in vivo*, 143B/Luc cells stably expressing APLN short hairpin RNA (shRNA) were established. Western blot results showed that APLN and PLOD2 protein expression was significantly inhibited in 143B/shAPLN-Luc cells stably expressing APLN shRNA compared with 143B/Luc control cells (Figure 8A-B) and cell migration was dramatically reduced (Figure 8C). To understand the *in vivo* effects of APLN knockdown in lung metastasis, 143B/Luc cells or 143B/shAPLN-Luc cells were injected into the lateral tail vein of each mouse, and tumor metastasis was monitored by bioluminescence imaging. APLN knockdown significantly inhibited the development of lung metastasis (Figure 8D-E). The mice were sacrificed at 9 weeks after the injections. *Ex vivo* imaging of the lungs showed that mice injected with 143B/Luc cells were significantly more likely to have lung metastasis than mice injected with 143B/shAPLN-Luc cells (Figure 8F-G). IHC staining indicated that the levels of APLN and PLOD2 protein expression were manifestly increased in the 143B/Luc-injected mice group compared with the 143B/shAPLN-Luc-injected mice (Figure 8H-J). These results confirm that inhibition of APLN diminishes osteosarcoma metastasis to the lung.

## Discussion

Osteosarcoma is a highly malignant tumor that exhibits high proclivity for a local invasion of bone and metastasis of the lung 54. Metastasis is the chief cause of death in osteosarcoma 55 and it is therefore necessary to clearly understand the molecular mechanisms underlying metastatic osteosarcoma for the development of effective therapeutic interventions. Our results found that APLN increases the expression of PLOD2 in osteosarcoma tissue, and thereby promotes osteosarcoma cell migration and induces osteosarcoma metastasis. Our investigations also revealed that the levels of APLN and PLOD2 expression are positively associated with tumor staging in patients with osteosarcoma. APLN induced the upregulation of PLOD2 expression via the Hippo signaling pathway and by upregulating the hsa_circ_0000004/miR-1303 axis. APLN appears to be an appropriate therapeutic target for osteosarcoma.

APLN, an adipokine secreted from adipose tissue, plays critical roles in many human cancers 56. Levels of *APLN* mRNA expression are higher in non-small cell lung cancer tissue than those in normal lung tissue and upregulation of APLN protein expression is related to poor overall survival of patients 26. APLN has previously been shown to stimulate tumor metastasis. APLN promotes the migration of gastric cancer cells 31, while in lung adenocarcinoma, apelin-13 (a 13-amino acid oligopeptide that serves as the ligand for the APLN receptor) enhances cancer cell migration via phosphorylation of the PAK1-cofilin signaling pathway 30. Apelin-13 induces breast cancer cell proliferation and invasion through the ERK1/2/AIB1 signaling pathway 57. In addition, apelin-13 can induce lymph node metastasis in mice transplanted with APLN-overexpressing melanoma cells 58 and high levels of APLN expression in bladder tumor tissue are associated with a higher tumor stage and a higher probability of distant metastasis, as well as vascular invasion 59. In this study, we found that APLN enhances human osteosarcoma cell migration and the development of lung metastasis was inhibited in mice injected with APLN knockdown cells.

Metastasis is a key cause of cancer-associated deaths and includes a complex multistep process. The ECM has a crucial role at many stages during tumor progression, especially in metastatic tumors 17. The deposition of modified collagen in the ECM is a critical factor in the motility of tumor cells in many cancers 60. PLOD2, a collagen-modifying enzyme, is highly expressed in many cancer types such as bone 15, glioblastoma 13, and hepatocellular carcinoma 61. By affecting collagen cross-links surrounding tumors, PLOD2 is associated with cancer progression 62. PLOD2 can enhance the development of a fibrous microenvironment and thus promote cell survival and pulmonary metastasis in breast cancer 63. Previous report has demonstrated that upregulation of PLOD2 expression is associated with lymph node metastasis, lung metastasis, as well as poor outcomes in osteosarcoma 38. Our study is the first to explain the association between PLOD2 expression and tumor stages in osteosarcoma tissue. An inhibitor of PLOD2 (minoxidil) can suppress sarcoma metastasis 64. Similarly, our study shows that minoxidil suppresses APLN-induced promotion of PLOD2 expression, leading to the inhibition of osteosarcoma cell migration. Our evidence demonstrates that APLN enhances PLOD2-dependent metastatic osteosarcoma. Thus, PLOD2 is a promising molecular target for the treatment of osteosarcoma metastases.

Investigations involving circRNAs have revealed their crucial roles in numerous biological processes, such as the initiation, progression, and metastasis of cancer 65. Notably, circRNAs can act as miRNA sponges and thereby affect downstream target gene and protein expression in many different cancers 52. For instance, circNASP inhibits the availability of miR-1253 and thus promotes the expression of FOXF1 in osteosarcoma cells, stimulating their proliferative and invasive activities 66. Moreover, circRNA cSMARCA5 reduces hepatocellular carcinoma metastasis by sponging miR-17-3p and miR-181b-5p, and increasing TIMP3 67. Our study concentrated on the role of circ_0000004 upon the migration of osteosarcoma cells. We observed significant reductions in miR-1303 expression after APLN treatment. Moreover, we also demonstrated that circ_0000004 competitively binds to miR-1303, preventing the binding of miR-1303 to PLOD2 and thus increasing PLOD2 expression. The results indicate that circ_0000004 acts as a sponge of miR-1303 and thus facilitates APLN-induced osteosarcoma cell migration and increases PLOD2 expression.

Hippo signaling controls organ size, cell regeneration, and contributes to tumorigenesis 68. The dysfunction of MST1 and MOB1 in the canonical mammalian Hippo pathway promotes cancer metastasis 45, 46. In this study, we found that knockdown of MST1 and MOB1 inhibited PLOD2 expression. As a major downstream effector of the Hippo pathway, the activation of YAP regulates target genes that mediate osteosarcoma tissue growth and metastasis 69, 70. We found that inhibition of YAP expression significantly reduced levels of PLOD2 expression and osteosarcoma cell migration, while transfecting osteosarcoma cells with MST1 and MOB1 siRNAs significantly reduced APLN-induced promotion of YAP binding to the PLOD2 promoter region. The MST1/MOB1/YAP pathway is therefore involved in APLN-induced increases in *PLOD2* mRNA expression and cell migration. Dysregulation of the Hippo pathway is associated with many human diseases, including cancer 71. Various intracellular signals, including inflammation, oxidative damage and growth factors, regulate the functions of YAP and TAZ 72, 73. Dephosphorylated YAP translocates to the nucleus and thereby activates genes involved in cell proliferation 74. Knockdown of the regulator of G-protein signaling 12 (*RGS12*) gene expression in osteosarcoma cells prevented YAP phosphorylation and inhibited lung metastasis 70. In our study, we found that stimulating cells with APLN prevented YAP phosphorylation, YAP-14-3-3 binding and osteosarcoma cell migration. Transfecting the cells with siRNAs silenced the expression of MST1 and MOB1, which significantly inhibited YAP translocation into the nucleus. The underlying mechanism of this process has yet to be clarified.

Several studies have indicated a link between the Hippo pathway and circRNAs, where circRNAs perform as competing endogenous RNAs (ceRNAs) to regulate miRNA functions 75. For example, YAP, TEAD1, and the mutant p53 complex increases circPVT1 expression, inhibiting the expression of miR-497-5p and thereby enhance head and neck squamous cell carcinoma proliferation 76. A limitation of our research is that we have not determined any link between the Hippo signaling pathway and hsa_circ_000004/miR-1303 axis in osteosarcoma. Further research into this aspect is necessary.

Finally, some limitations should be noted in this study. Firstly, the study results could have been strengthened statistically by using more osteosarcoma cell lines. Ideally, we would have used the Biomax OS208a tissue microarray containing 22 cases of human osteosarcoma and 9 adjacent normal tissue samples, but this array is out of stock. We could therefore only use the OS804D array, which contains two stage IVB osteosarcoma tissue samples (showing significantly high levels of APLN expression). Our search for more such samples identified the OR803 array, which also contains two stage IVB osteosarcoma tissue samples, but the clinicopathologic data (age, sex and pathology diagnoses) appear to match those of the OS804D samples. Thus, our study was unable to analyze more than two stage IVB osteosarcoma tissue samples. Moreover, the two cell lines used in this study behave differently in humans, with one (143B) being more likely to cause metastases. Secondly, although our data strongly suggest that APLN promotes PLOD2-mediated cell migration in OSCC* in vitro*, we cannot exclude the possibility that APLN also promotes the activities of other factors that influence metastasis, such as vascular endothelial growth factor, integrin and epithelial-mesenchymal transition (EMT)-associated proteins. Thirdly, the injection of osteosarcoma cells into the tail vein for initiating metastases in the lung tissue may ignore the complexity of the metastasis process that depends upon the primary tumor, the tumor microenvironment and secondary organs.

**Figure 9:**
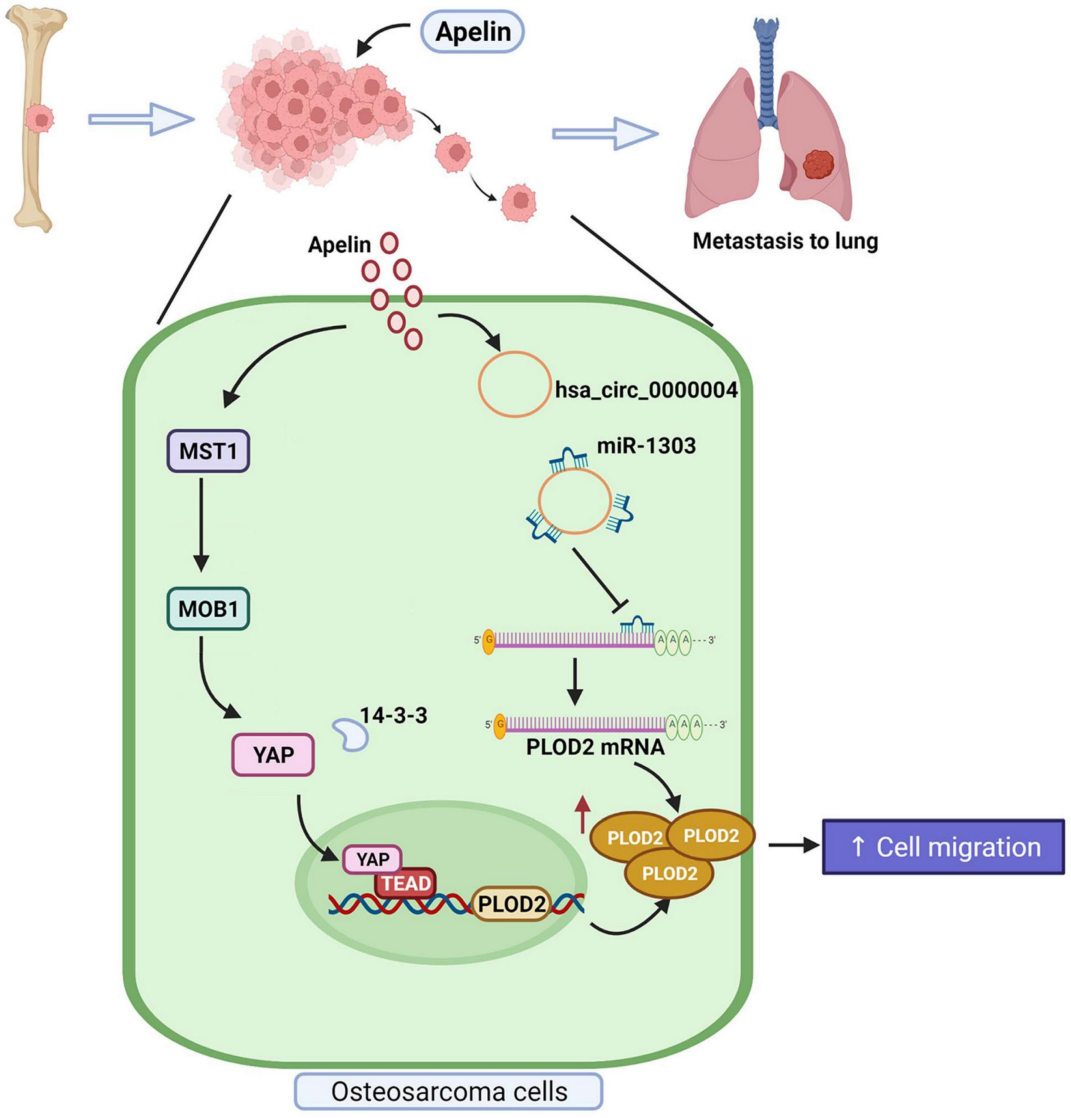
**Schema illustrates the involvement of APLN in osteosarcoma metastasis.** APLN facilitates PLOD2-dependent migratory activities of osteosarcoma cells through the MST1, MOB1 and YAP signaling cascades and the miR-1303/hsa_circ_0000004 axis.

In conclusion, our study demonstrates that APLN promotes PLOD2 expression and the migration of human osteosarcoma cells via the MST1, MOB1 and YAP signaling cascades and hsa_circ_0000004/ miR-1303 axis (Figure 9). APLN appears to be a promising target in metastatic osteosarcoma.

## Supplementary Material

Supplementary materials and methods, figures.
