# Supplementary material for: Apelin promotes osteosarcoma metastasis by upregulating PLOD2 expression via the Hippo signaling pathway and hsa_circ_0000004/miR-1303 axis
Source: Int J Biol Sci. 2023 Jan 1;19(2):412–25. doi: 10.7150/ijbs.77688 (PMC9830518; doi:10.7150/ijbs.77688)
Supplement: Supplementary file 1 — Supplementary materials and methods, figures. [file ijbsv19p0412s1.pdf]

Supplementary file

Materials and Methods

Materials

APLN recombinant protein was bought from MyBioSource (San Diego, CA, USA). APLN antibody was bought from Aviva Systems Biology (San Diego, CA, USA) and PLOD2 antibody was obtained from R&D systems (Minneapolis, MN, USA); antibodies specific for mammalian Sterile 20-like kinase 1 (MST1; Catalog No: 3682S), monopolar spindle-one-binder protein 1 (MOB1; Catalog No: 13730S), Yes-associated protein (YAP; Catalog No: 14074), phospho-MST1 (Catalog No: 49332S), phospho-MOB1 (Catalog No: 8699S), phospho-YAP (Ser127; Catalog No: 13008S) were supplied by Cell Signaling Technology (Danvers, MA, USA). Minoxidil, XMU MP1, verteporfin, and (R)-PFI 2 hydrochloride were purchased from Tocris Bioscience (Bristol, UK). Small interfering RNAs (siRNAs) against MST1 (Catalog No: sc-39249), MOB1 (Catalog No: sc-94744), YAP (Catalog No: sc-38637) and signal transducer and activator of transcription 3 (STAT3) inhibitor VI S3I-201 (Catalog No: sc-204304) were bought from Santa Cruz Biotechnology (Santa Cruz, CA, USA). The PLOD2 siRNA (Catalog No: L-004285-01-0005) was purchased from Dharmacon Research (Lafayette, CO, USA). The hsa_circ_0000004 siRNA 1 (sequence: 5’-GGCAGGCCACGGCATATGCCT-3’), siRNA 2 (sequence: 5’-GTCCTGGCAGGCCACGGCATA-3’) and siRNA 3 (sequence: 5’-TCCTGGCAGGCCACGGCATAT-3’) were purchased from MDBio Inc. (Taipei, Taiwan). Lipofectamine 2000 and microRNA (miR)-1303 mimic were acquired from Thermo Fisher Scientific (Waltham, MA, USA). Reporter lysis buffer was supplied by Promega (Madison, WI, USA). A commercial human osteosarcoma tissue array (OS804d) was bought from US Biomax, Inc. (Rockville, MD, USA). Normal bone and osteosarcoma tissue samples were collected from 7 patients undergoing surgical

resection in China Medical University Hospital. This study was conducted according to the Declaration of Helsinki guidelines after obtaining approval from the Institutional Review Board of China Medical University Hospital. Rapamycin (Catalog No: R0395), pyrrolidine dithiocarbamate (PDTC; Catalog No: P8765), hypoxia inducible factor-1α inhibitor (Catalog No: 400083) and all other chemicals were obtained from Sigma-Aldrich (St. Louis, MO, USA).

Migration assay

A cell migration assay was performed using Transwell plates (Costar, NY; pore size, 8 mm). Before performing this assay, the cells were pretreated with different concentrations of inhibitors for 30 min or transfected with siRNAs for 24 h. A total of 200 mL of serum-free medium containing approximately 10^4 cells was placed in the upper compartment; 300 ml of DMEM 2% FBS was placed in the lower compartment. After incubating the plates with 5% CO_2 for 24 h, the cells were fixed in 3.7% formaldehyde for 15 min and stained with 0.05% crystal violet in phosphate-buffered saline (PBS) for 15 min. Cells on the upper side of the filter were removed with cotton swabs, and the filters were washed with PBS. Migrated cells were counted under a microscope.

Western blot assay

Cell lysates were prepared in RIPA buffer containing a protease inhibitor cocktail. Proteins were separated using sodium dodecyl sulfate-polyacrylamide gel electrophoresis and transferred to Immobilon polyvinyl difluoride membranes (Millipore, MA, USA). The membranes were blocked in 5% non-fat milk at room temperature for 1 h, then incubated with primary antibodies PLOD2, p-MST1, MST1, p-MOB1, MOB1, p-YAP, YAP, 14-3-3, and β-actin (1:1000) for 1 h at room temperature.

After undergoing 3 washes with Tris-buffered saline and 0.05% Tween 20, the membranes were incubated with a donkey anti-rabbit or anti-mouse peroxidase-conjugated secondary antibody (1:3000) for 1 h at room temperature. The blots were developed using enhanced chemiluminescence and imaged using a Fujifilm LAS-3000 chemiluminescence detection system (Fujifilm, Tokyo, Japan).

Reverse transcription-quantitative PCR (RT-qPCR) assay

Total RNA was isolated from osteosarcoma cells using TRIzol reagent and RNA concentrations were quantified using a NanoVue Plus spectrophotometer (GE Healthcare Life Sciences; Pittsburgh, PA, USA). Reverse-transcription of total RNA into cDNA was performed using the M-MLV RT kit (Thermo Fisher Scientific; Waltham, MA, USA), the Mir-X™ miRNA First-Strand Synthesis kit (Clontech; Mountain View, CA, USA) and the Primescript™ RT reagent (TaKaRa, CA, USA). The qPCR assay was performed using StepOnePlus (Applied Biosystems).

Immunohistochemistry (IHC) staining

A human osteosarcoma tissue array (OS804d) was purchased from US Biomax, Inc. (Rockville, MD, USA) and mouse lung tissues were deparaffinized in xylene and rehydrated in ethanol at decreasing concentrations. Heat-induced antigen retrieval was performed on all sections in 0.01 M sodium citrate buffer (pH 6) at 95°C for 10 min. Human APLN and PLOD2 antibodies were used at a dilution of 1:200 and incubated at 4°C overnight. Antibody-binding signals were measured using the NovoLink Polymer Detection System (Leica Microsystems) and visualized using 3-3'-diaminobenzidine tetrahydrochloride as a substrate for color development. The sections were counterstained with hematoxylin. IHC results were scored using MacBiophotonics ImageJ software. The staining intensity was graded as 0 (no staining), 1 (<25% of

positive staining), 2 (25-49% of positive staining), 3 (50-74% of positive staining), and 4 (75-100% of positive staining).

Cell transfection

Osteosarcoma cells were seeded in 6-well dishes, then transfected with control, PLOD2, MST1, MOB1, or YAP siRNAs using Lipofectamine 2000 transfection reagent, according to the manufacturer's recommendations. The cells were then incubated with APLN (3 ng/mL) for 24 h. The cell extracts were prepared and used for qPCR or Western blot analysis.

APLN shRNA was transfected into 143B and 143B/Luc cells using jetPEI transfection reagent. After 48 h, stable transfectants were selected in puromycin at a concentration of 2 μg/mL. Thereafter, the selection medium was replaced every 4 days. After 2 weeks of selection in puromycin, clones of resistant cells were isolated.

RNA pull-down assay

The 143B cells were transfected with biotinaylated WT-miR-1303 by Lipofectamine 2000 for 24 h. The cells were collected and washed with PBS, then lysed in SDS lysis buffer for 10 min. Cell lysate was incubated with Dynabeads™ M-280 streptavidin (Invitrogen, Carlsbad, CA, USA) at 4°C. After 24 h, the beads were washed twice with lysis buffer, 3 times with low-salt buffer, and once with high-salt buffer, respectively. The bound RNA was purified with TriZol and qPCR was performed to detect the expression of hsa_circ_0000004.

Figures

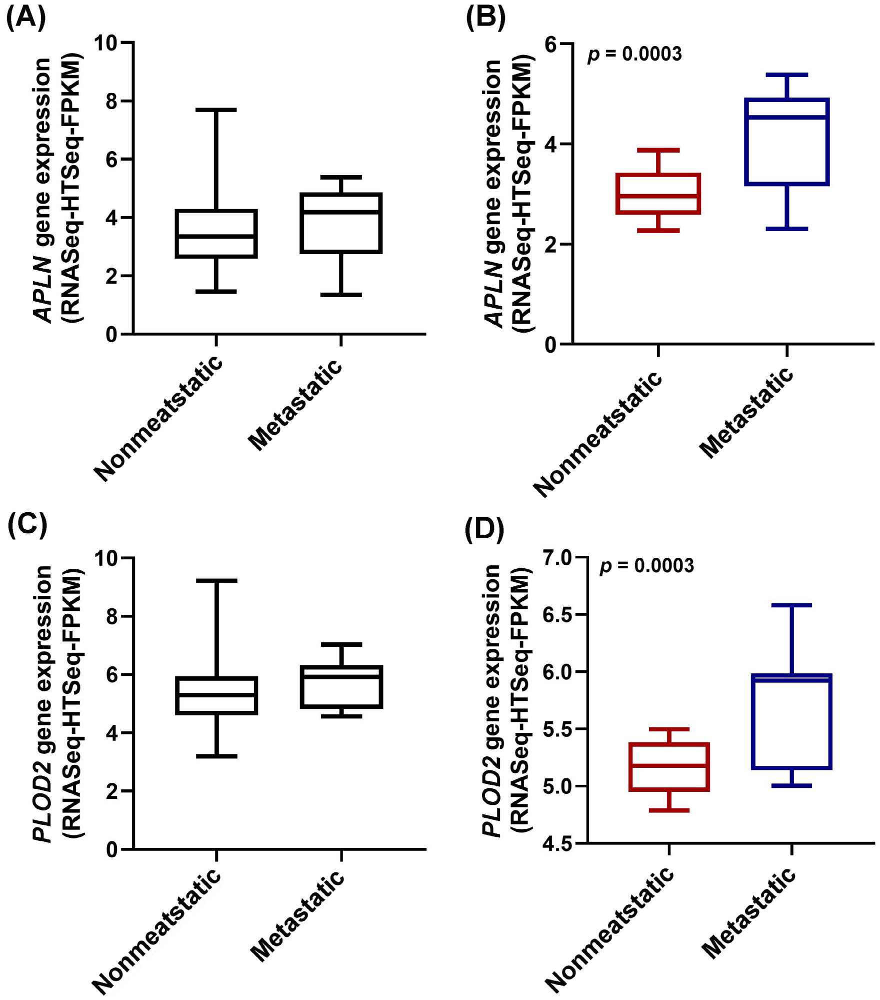

Figure S1. Positive associations between *APLN* and *PLOD2* expression and nonmetastatic or metastatic osteosarcoma tissue. *APLN* and *PLOD2* mRNA expression in nonmetastatic and metastatic tissues were analyzed using records from The Cancer Genome Atlas (TCGA) database. (A & C) All sample data. (B & D) 95% confidence intervals of (A & C) for the medians of all sample data.

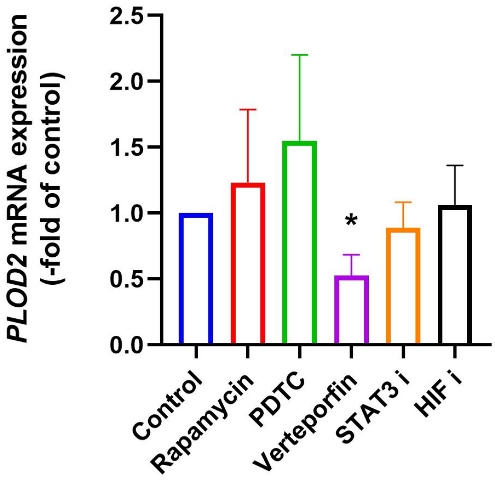

Figure S2. Hippo signaling is involved in APLN-induced *PLOD2* mRNA expression. 143B cells were incubated with 10 μM of rapamycin (mTOR inhibitor), 10 μM of PDTC (NF-κB inhibitor), 0.5 μM of verteporfin (inhibitor of YAP; the transcriptional co-activator of the Hippo pathway), 10 μM of STAT3 i (STAT3 inhibitor VI S3I-201), or 10 μM of HIF i (Hypoxia inducible factor-1α inhibitor) for 30 min, then treated with APLN (3 ng/ml) for 24 h. *PLOD2* mRNA expression was examined by qPCR.
